# Monolayer MoS_2_-Based Flexible and Highly Sensitive Pressure Sensor with Wide Sensing Range

**DOI:** 10.3390/mi13050660

**Published:** 2022-04-22

**Authors:** Dandan Xu, Ling Duan, Suyun Yan, Yong Wang, Ke Cao, Weidong Wang, Hongcheng Xu, Yuejiao Wang, Liangwei Hu, Libo Gao

**Affiliations:** 1 School of Mechano-Electronic Engineering, Xidian University, Xi’an 710071, China; ddxu@stu.xidian.edu.cn (D.X.); charlotte_duan@163.com (L.D.); yansuyun1018@163.com (S.Y.); xuhongcheng@stu.xidian.edu.cn (H.X.); yuejiwang4-c@my.cityu.edu.hk (Y.W.); 2 CityU-Xidian Joint Laboratory of Micro/Nano-Manufacturing, Xi’an 710071, China; 3 School of Advanced Materials and Nanotechnology, Xidian University, Xi’an 710171, China; yongwang@xidian.edu.cn; 4 School of Aerospace Engineering, Xiamen University, Xiamen 361102, China; 19920211151512@stu.xmu.edu.cn

**Keywords:** flexible electronics, flexible sensor, wearable sensor, iontronic pressure sensor, MoS_2_

## Abstract

Flexible pressure sensors play an important role in flexible robotics, human-machine interaction (HMI), and human physiological information. However, most of the reported flexible pressure sensors suffer from a highly nonlinear response and a significant decrease in sensitivity at high pressures. Herein, we propose a flexible novel iontronic pressure sensor based on monolayer molybdenum disulfide (MoS_2_). Based on the unique structure and the excellent mechanical properties as well as the large intercalation capacitance of MoS_2_, the prepared sensor holds an ultra-high sensitivity (*S*_max_ = 89.75 kPa^−1^) and a wide sensing range (722.2 kPa). Further, the response time and relaxation time of the flexible sensor are only 3 ms, respectively, indicating that the device can respond to external pressure rapidly. In addition, it shows long-term cycling stability (over 5000 cycles with almost no degradation) at a high pressure of 138.9 kPa. Finally, it is demonstrated that the sensor can be used in physiological information monitoring and flexible robotics. It is anticipated that our prepared sensor provide a reliable approach to advance the theory and practicality of the flexible sensor electronics.

## 1. Introduction

Flexible pressure sensors are widely used in human-machine interaction (HMI) [1,2,3], electronic skin (E-Skin) [4,5,6], and human health monitoring [7,8,9,10,11]. In particular, capacitive pressure sensors have attracted a broad attention with their merits of low power, excellent stability and fast response time. But building flexible capacitive pressure sensors with high sensitivity and wide sensing range has been a challenge when considered the sensitivity of conventional capacitive pressure sensors is typically relatively low.

Iontronic pressure sensors have recently attracted a lot of attention [12,13,14,15,16,17]. This type of interfacial capacitive sensor based on electrical double layer (EDL) capacitance has the advantages of ultra-high capacitance (μF cm^−2^) and high signal-to-noise ratio (SNR), which are not available with conventional flat plate capacitive sensor. The EDL performance depends on many items, among which the electrode materials play a most important role. Currently reported sensitive materials for preparing iontronic pressure sensor is categorized into metal-based electrode materials [18,19], carbon-based materials [7], metal oxide indium tin oxide (ITO) [20], and MXene [21]. Metal electrodes typically exhibit smaller specific surface area and reduced EDL capacitance. In the case of carbon materials, the electrodes are thicker, mechanically prone to fracture and have longer ion transport paths, while MXene faces problems such as oxidation. Therefore, the deep research on new materials for iontronic pressure sensor and the further study of their sensing mechanisms are important for the development of flexible iontronic sensors.

As a new two-dimensional material, molybdenum disulfide (MoS_2_) have attracted a lot of attention in recent years, and it has been widely used in supercapacitors, microelectronic devices, and batteries because of its rich elemental composition, wide band gap (1.29−1.90 eV), high electron mobility, excellent thermal properties and good mechanical properties [22,23,24]. Therefore the MoS_2_ has the potential to be used as an electrode material in iontronic pressure sensors. However, the study on the monolayer MoS_2_-based flexible iontronic pressure sensor are rarely reported.

Therefore, in order to solve the problem of sensitivity and sensing range balance of the flexible pressure sensor, we propose a novel iontronic pressure sensor based on MoS_2_ monolayer. The monolayer MoS_2_/gold composite electrode was obtained by wet transfer strategy. And the iontronic pressure sensor was assembled by the composite electrode and microstructural ionic film. Thanks to the excellent mechanical-electrical properties of MoS_2_ and unique layered structure, the prepared sensor shows an unprecedented ultra-high sensitivity (*S*_max_ = 89.75 kPa^−1^, *S*_min_ = 10.24 kPa^−1^) and a wide sensing range (722.2 kPa). The response time and relaxation time of the sensor sensor are both 3 ms, respectively. Moreover, the possible sensing mechanism of the sensor is proposed. Finally, the application of this novel iontronic pressure sensor is carefully demonstrated.

## 2. Materials and Methods

### 2.1. Preparation of MoS_2_/Au Composite Electrode

The Au/PI (polyimide) was prepared by magnetron sputtering. A thin 100 nm gold layer was sputtered on the cleaned PI film. A monolayer MoS_2_ grew on the Si/SiO_2_ (Silion/Silicon dioxide) wafer (SiO_2_ layer thickness, 300 nm) by chemical vapor deposition (CVD) method. A PMMA (polymethyl methacrylate) film was covered on the MoS_2_ layer as a supporting protective layer by spin-coating method (3000 r min^−1^, 10 s), and dried completely at 100 °C for 10 min. Then, the samples were placed in a KOH (potassium hydroxide) solution (1 mol L^−1^) with a heating temperature of 90 °C to etch the growth substrate to separate the PMMA/MoS_2_ from the Si/SiO_2_ substrate. After the sample was completely separated from the substrate, PMMA/MoS_2_ was transferred to deionized water and soaked several times to remove the residue KOH solution, and then PMMA/MoS_2_ was transferred to the Au/PI electrode. Finally, the PMMA film was removed using acetone, and the electrode was cleaned with isopropyl alcohol and deionized water several times to remove the residual acetone.

### 2.2. Preparation of PVA/H_3_PO_4_ Film with Pyramid Microstructure

1 g of Polyvinyl Alcohol (PVA) was mixed with deionized water (10 mL) under serious stirring at 85 °C for one hour until completely dissolved to a transparent state. Then the 85% H_3_PO_4_ solution (2 mL) was added to the prepared PVA solution drop by drop, and then stirring continued for half an hour at 90 °C to make the uniform dispersion. Finally, the prepared gel electrolyte was evenly coated on the mask template with pyramidal microstructure and the thickness was controlled at 100 μm by spin coating. After the film was dried at 60 °C for one hour, the film can be peeled off from the substrate.

### 2.3. Fabrication of Flexible Pressure Sensor

The prepared gold electrode with a monolayer MoS_2_ on PI substrate, the poly (ethylene terephthalate) PET spacer layer, the PVA/H_3_PO_4_ ionic film with upside-down pyramid microstructure are stacked in a “sandwich” configuration PET with a thickness of about 100 μm was employed as the spacer, and finally 3M tape was used to ensure the fully integrated encapsulation of the device.

### 2.4. Material and Structural Characterization

Field emission scanning electron microscopy (FESEM, Quanta 450, 20 kV) were used to characterize the structure and morphology information. Raman spectroscopy were measured by a Renishaw inVia Raman spectrometer (Renishaw, Britain, UK). For each test on MoS_2_, three samples were prepared and five points on each sample were measured.

### 2.5. Electrical and Mechanical Characterization

For the electromechanical characterization, the variable capacitance can be recorded by LCR impedance analyzer (Tonghui, TH 2827A, Changzhou, China) and IM3536 LCR meter (HIOKI, Taiwan, China) linkage test. The CV curves also were shown on the electrochemical working station (CHI 760E, Chen Hua, Shanghai, China) under the applied pressure. For featuring cyclic stability, the device was tested on a mechanical testing machine (Zhi Qu, 990B, Dongguan, China) with the pulling speed of 5 mm min^−1^.

## 3. Results and Discussion 

**Figure 1:**
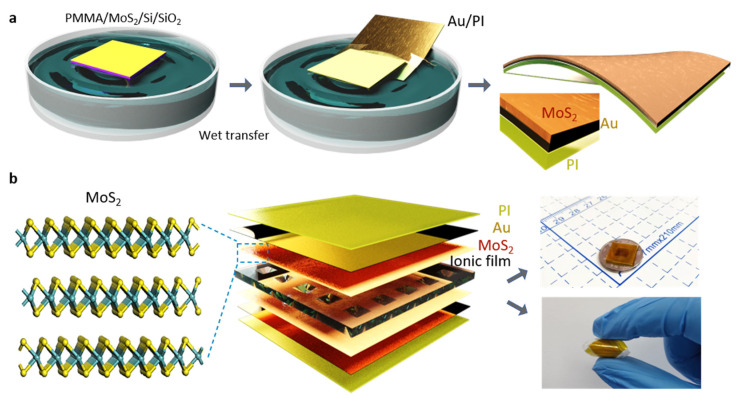
Preparation and schematic diagram of iontronic pressure sensor. (**a**) Wet transfer process of MoS_2_ monolayer. (**b**) Digital optical image and flexible feature of iontronic pressure sensor and the corresponding diagram layout of each layer in the sensor.

MoS_2_ possess excellent intercalating pseudocapacitive and EDL properties for charge storage. And it has been shown that MoS_2_ has faster electron transfer capability than oxide and higher theoretical specific capacitance than graphene when used as an electrode material. Therefore, in this study, a monolayer MoS_2_ is introduced as the electrode to improve the capacitance and further boost the sensitivity of the sensor. The monolayer MoS_2_ was transferred by wet transfer method (Figure 1) on an Au/PI film. The assembled device is shown in Figure 1b. The size of the prepared sensor is 1 × 1 cm^2^, which is small compared with a coin, demonstrating its portable and flexible feature. Noted that the microstructural ionic film was beneficial to further improve the sensitivity of the sensor, which has been demonstrated by previous reports [25].

**Figure 2:**
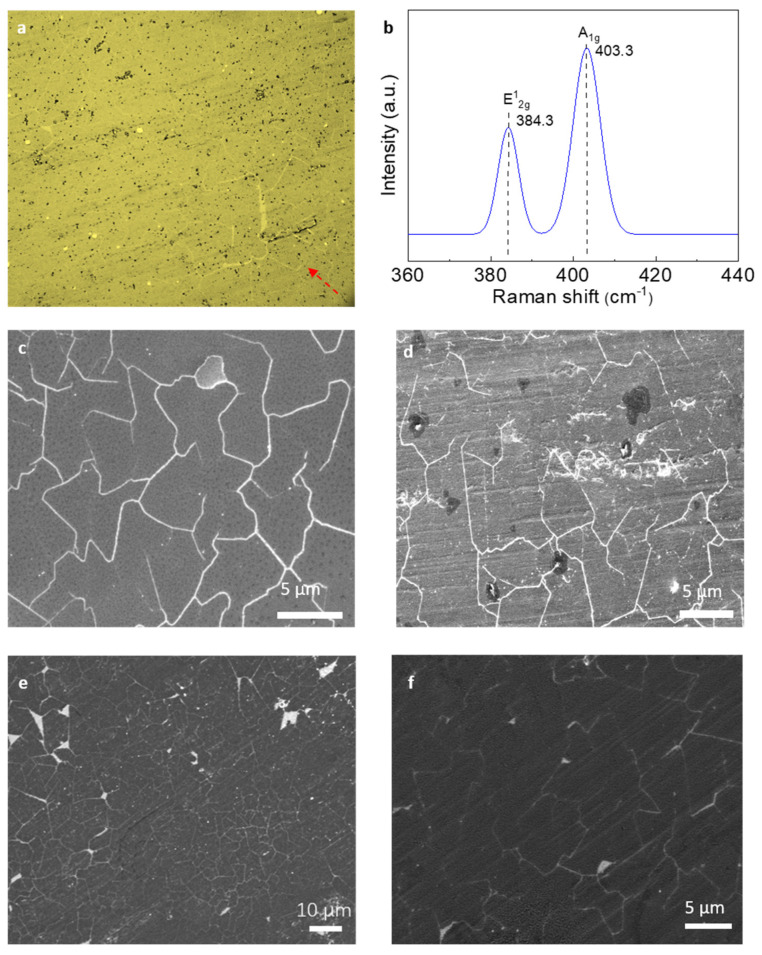
Characterization of the sensor electrodes for flexible sensors. Scanning electron microscopy (SEM) images of monolayer MoS_2_. (**a**) Optical image of the monolayer MoS_2_ on Au/PI film. (**b**) Raman data of the monolayer MoS_2_. (**c**,**d**) SEM of the monolayer MoS_2_ before and after transfer process. (**e**,**f**) SEM image after 5000 cycle stability test.

The monolayer MoS_2_ was transferred onto the Au/PI electrode by wet transfer method [26], and the optical microscope of the electrode is shown in Figure 2a. From which it can be observed that the large and continuous monolayer MoS_2_ on the Au/PI film showed good integrity and only a very few cracks existed due to etching when the support layer was removed during the transfer process. The Raman results further certified the MoS_2_ property. The number of layers of the sample can be determined according to the wave number difference between E^1^_2g_ and A_1g_.from Raman spectra of monolayer MoS_2_. Wave number difference of bulk material is 26 cm^−1^, while the wave number difference between A_1g_ and E^1^_2g_ in Figure 2b is 19 cm^−1^. Hence, the preparative sample of MoS_2_ can be regarded as monolayer with the thickness of 6.15 Å [27,28]. Further, SEM of the monolayer MoS_2_ before and after the transfer was performed in Figure 2c,d. It can be observed that the monolayer MoS_2_ on the Si/SiO_2_ substrate is large area continuous growth without clear defect. Also, as shown in Figure 2d, the monolayer MoS_2_ can still be observed as a continuous boundary which is similar to that before transfer to the target substrate by the wet transfer method. This further indicates that the monolayer MoS_2_ obtained by the wet transfer method has a high integrity with few significant cracks, which lays the foundation for the preparation of the sensor electrode. Finally, SEM of the monolayer MoS_2_ after 5000 cycle test was performed in Figure 2e,f. 

**Figure 3:**
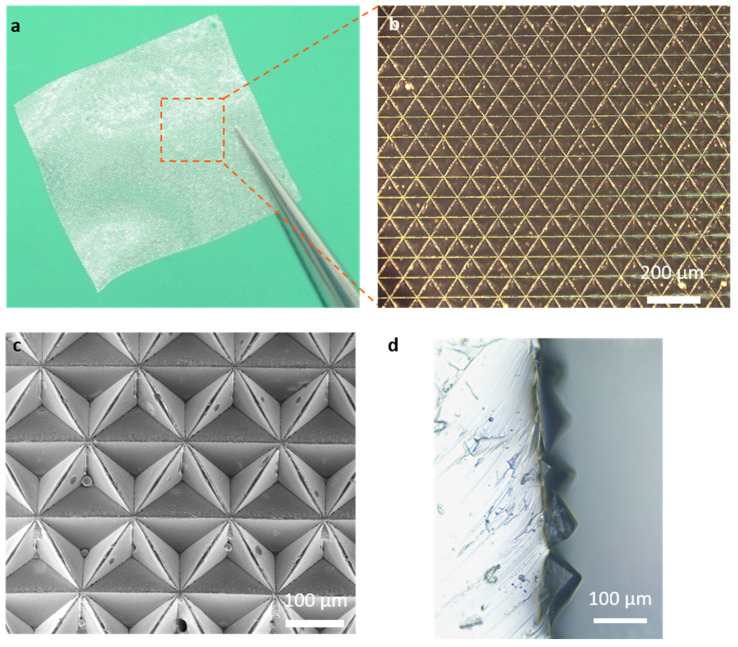
Characterization of the ionic film for flexible sensors. (**a**) Low and (**b**) high magnification of the optical image of the ionic film. (**c**) SEM image of the ionic film. (**d**) Cross-sectional view of the ionic film.

The digital optical image of the ionic membrane is shown in Figure 3a, which exhibits excellent flexibility. The microstructure of the inverted pyramid is clearly visible (Figure 3b). The prepared PVA/H_3_PO_4_ ionic membrane was further characterized by SEM, as shown in Figure 3c. It can be clearly seen that the microstructure of the prepared ionic membrane is well established, exhibiting an inverted pyramidal structure with each size of about 100 μm. The cross-sectional structure of the ionic membrane is shown in Figure 3d, which shows that the membrane has a distinct pyramidal protrusion structure with a height of about 70 μm. The microstructure has been demonstrated to significantly improve the sensitivity of the flexible sensor. When the microstructure is compressed, due to the initial mechanically non-stationary state, it is easy to deform thus causing a rapid increase in the contact area between the electrode and the ionic membrane, thus increasing the capacitance and sensitivity of the sensor.

**Figure 4:**
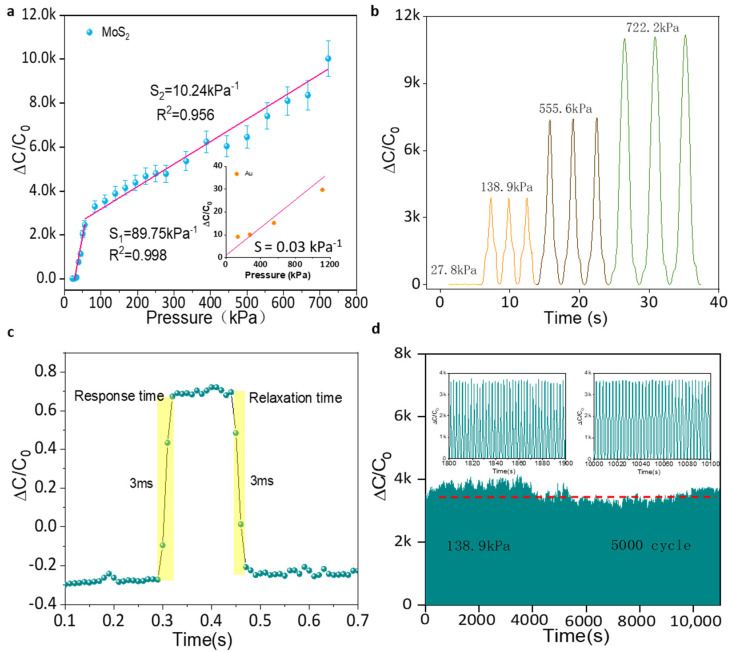
The performance of the flexible sensor. (**a**) Capacitance variation of the sensor under various pressure. (**b**) Continuous pressure test of the sensor. (**c**) Response and relaxation time of the flexible sensor. (**d**) Durability test of the sensor under applied pressure of 138.9 kPa for 5000 cycles.

The performance of the sensor is investigated as shown in Figure 4. The maximum sensitivity (*S*_max_) of the MoS_2_-based iontronic pressure sensor is 89.75 kPa^−1^ up to 55.6 kPa, and the minimum sensitivity(*S*_min_) is 10.24 kPa^−1^ in the range of 55.6 kPa to 722.2 kPa, which is superior to our previous work and other works [29,30,31,32,33,34]. Also, an iontronic pressure sensor with the same structure but without the MoS_2_ layer was prepared as a control group. The results showed that its sensitivity was 0.031 kPa^−1^, which demonstrated the important role of the MoS_2_ layer. The increased capacitance is derived from the unique layer structure, huge specific surface area and pseudocapacitance characteristics. 

The stability of the sensor subject to the same pressure is also one of the important factors. The sensor was tested under four different continuously pressure of 27.8 kPa, 138.9 kPa, 555.6 kPa, and 722.2 kPa respectively, as shown in Figure 4b. The signal of the sensor is constant for each test, and can be rapidly recovered after the force is removed. The stable and proportional electrical signal output at different pressures indicates that the sensor can maintain high stability with small signal drift. To evaluate the dynamic response speed of the pressure sensor, a weight of 1 g (equivalent pressure ~0.01 N) was gently placed on the pressure sensor followed by a quick release revealing a 3 ms response time and relaxation time in Figure 4c. This indicates that the prepared sensor can respond quickly to the applied external stimulus and has a good dynamic response. Even after continuous pressure of 138.9 kPa for 5000 cycles, as shown in Figure 4d, the sensor still maintained a stable signal output with no significant attenuation, demonstrating its long-term use application. Small cracks were found on the surface of monolayer MoS_2_ but they did not affect final signal response in Figure 4d.

**Figure 5:**
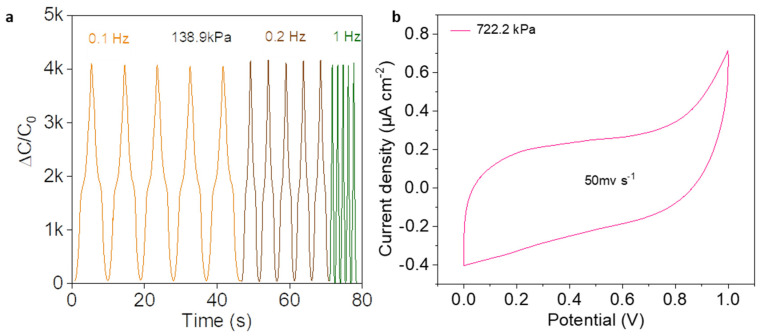
(**a**) Capacitance variation of the sensor under various frequencies. (**b**) CV curve of the sensor at a scan rate of 50 mV s^−1^.

Also, the sensor was further tested at different frequencies (0.1, 0.2 and 1 Hz) at the same pressure (138.9 kPa). As shown in Figure 5a, the sensor maintained a stable signal at various frequency, demonstrating that the sensor’s excellent dynamic response. Importantly, the CV curve of the sensor under 722.2 kPa was investigated to find the deep sensing mechanism. As shown in Figure 5b, the CV curve is not ideally rectangular, and shows a small peak in redox, which is further evidence of the pseudo-capacitance effect as described above.

**Figure 6:**
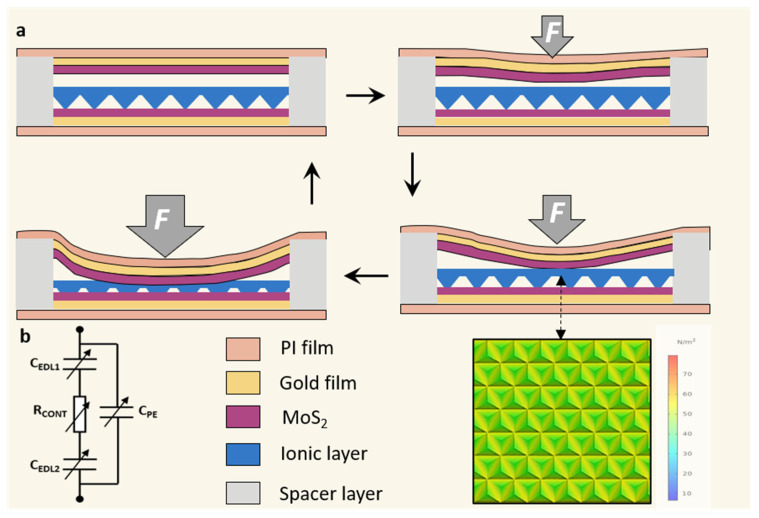
Schematic illustration of the working mechanism of the sensor. (**a**) Sensing mechanism of the iontronic pressure sensor and the stress concertation of the microstructure. (**b**) Corresponding capacitance variation of the sensor.

Based on the above, the superior performance of the sensor has been demonstrated and its sensing mechanism was revealed as shown in Figure 6. In this study, in addition to improving the sensitivity by MoS_2_ layer, a spacer layer is also introduced between the ionic film and the electrode (Figure 6a). Initially, the capacitance is extremely small due to the presence of the spacer layer. When the sensor is subjected to pressure, the upper electrodes layer was bent due to stress and the distance between the two electrodes becomes narrow, resulting in a larger capacitance (C_PE,_ Figure 6b). Further, when the force continues to be applied, the electrodes began contact with the ionic membrane (C_EDL1_), causing a rapid increase in EDL capacitance. With further increase in force, the contact area between the microstructure of the ionic membrane and the lower electrode further increases and the capacitance further becomes larger (C_EDL2_), leading to higher capacitance changes. In addition, it is obvious from the simulation that the deformation of the microstructure is mainly concentrated in the middle part of the microstructure rather than the tip of the structure, which may be due to the unique shape of the microstructure. It is also confirmed that the microstructure can improve the capacitance of the sensor.

**Figure 7:**
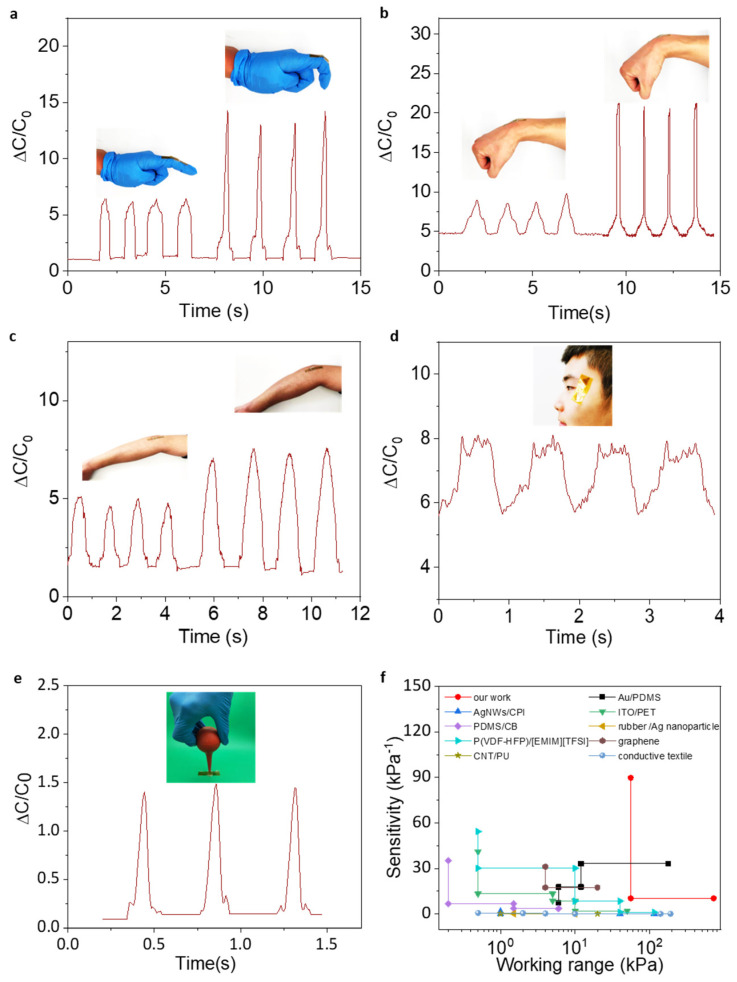
Practical application of the flexible iontronic sensor for montion monitoring. Capacitance variations of the sensor during (**a**) finger (**b**) wrist (**c**) arm bending and (**d**) eye blinking. (**e**) Capacitance response output of gas leak monitoring, and the image is shown in the inset. (**f**) Comparison of the sensitivity of our pressure sensor with existing capacitive sensors.

To investigate the application of the iontronic pressure sensor, the sensor was mounted on the human body to detect the movement. As shown in Figure 7a, with the rapid change of the finger to a small angle, the signal output of the sensor is consistent each time, indicating that the sensor can accurately recognize the bending of the finger. Subsequently, with a rapid change of the finger to a large angle (about 90°), the signal output amplitude increases significantly compared to the previous small angle change, suggesting that the sensor can accurately recognize different angle changes of the finger. In addition to the finger, the sensor can also detect wrist and arm bending as well as eye blinking (Figure 7b−d). These above demonstrated that the sensor has the ability to detect human motion and has important applications in the health rehabilitation of the human body. To simulate the scene of gas leak monitoring, an aurilave was used to imitate airflow. Under subtle signals, the sensor response was captured. Figure 7e has shown the variation of capacitance over time, which demonstrated that stable output can also be achieved for subtle signals. As summarized in Figure 7f [35,36,37,38,39,40,41,42,43], our Monolayer MoS_2_-based iontronic pressure sensor shows an incomparably high sensitivity and an ultrabroad work range of pressure, out-performing existing capacitance pressure sensor.

**Figure 8:**
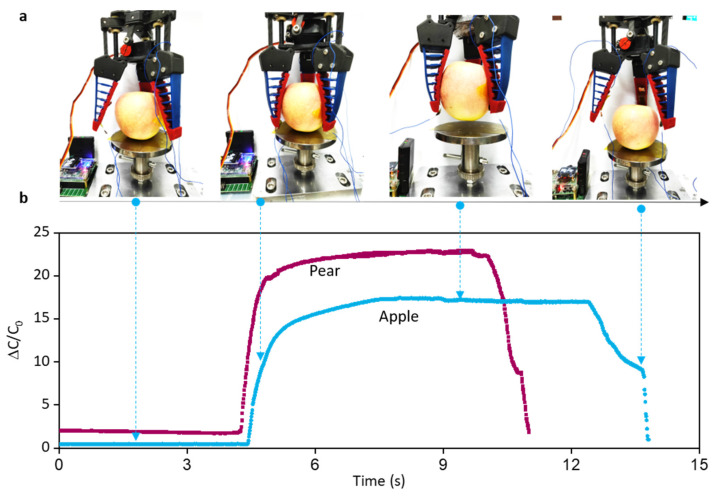
Application of the sensor on flexible robotics. (**a**) The process to grasp and release apple; (**b**) Corresponding steps for the soft manipulator to grasp and release the apple and pear sample.

Further, we directly mounted the flexible sensor on the flexible manipulator and use the manipulator to grasp objects of different weights. In this study, pears and apples of different weights but similar shapes for comparison. As shown in Figure 8, the capacitance change trend of both is the similar, but the magnitude is different, which is due to the different magnitude of force applied by the manipulator when grasping two different weight objects. This successfully demonstrates that the prepared sensor has the ability to recognize the weight of the target and can be used as a tactile sensor to fix on the flexible manipulator to recognize the mass of the object.

## 4. Conclusions

In summary, a highly sensitive iontronic pressure sensor based on EDL sensing mechanism was designed and prepared. The electrodes based on a monolayer MoS_2_ were successfully prepared by the wet transfer method, and the PVA/H_3_PO_4_ ionic membrane with inverted pyramid microstructure was beneficial to enhancing the sensitivity. The pressure sensor showed a high sensitivity of 89.75 kPa^−1^ in the sensing range of 722.2kPa. Additionally, the sensor exhibited a short response time and relaxation time. Through analysis, the unique structure-induced high capacitance, the rationally designed structure such as the microstructural ionic film and device configuration are all beneficial to enhancing the sensitivity and sensing range. Finally, it is verified that the sensor has a great potential for motion detection and flexible robotics applications. Our sensor design makes a novel strategy for flexible sensor electronics and will inspire more committee focus on 2D materials-based sensor.

The next research should focus on the following items.

Firstly, the effect of different layers of MoS_2_ on the performance of the sensor and the mechanism of the effect should be investigated.

Secondly, the sensors should be further miniaturized to prepare higher integrated sensors such as the sensor arrays with a high sensitivity to achieve a large-scale application.

## Data Availability

The data presented in this study are available on request from the corresponding author.
